# Chronic Achilles Tendon Rupture

**DOI:** 10.2174/1874325001711010660

**Published:** 2017-07-31

**Authors:** Nicola Maffulli, Alessio Giai Via, Francesco Oliva

**Affiliations:** 1 Department of Musculoskeletal Disorders, School of Medicine and Surgery, University of Salerno, Salerno, Italy; 2 Queen Mary University of London, Barts and the London School of Medicine and Dentistry, Centre for Sports and Exercise Medicine, Mile End Hospital, 275 Bancroft Road, London E1 4DG, England; 3 Department of Orthopaedic and Traumatology, University of Rome “Tor Vergata”, School of Medicine, Viale Oxford 81, 00133 Rome, Italy

**Keywords:** Achilles tendon, Chronic ruptures, Minimally-invasive surgery, Neglected injury, Tendon ruptures, Tendon transfer

## Abstract

**Background:**

The Achilles tendon, the largest and strongest tendon in the human body, is nevertheless one of the tendons which most commonly undergoes a complete subcutaneous tear. Achilles tendon ruptures are especially common in middle aged men who occasionally participate in sport. Even though Achilles tendon ruptures are frequent, up to 25% of acute injuries are misdiagnosed, and present as chronic injuries.

**Methods:**

This is a review article about diagnosis and management of chronic Achilles tendon ruptures. Minimally invasive Achilles tendon reconstruction is discussed.

**Results:**

The optimal surgical procedure is still debated, however, less invasive peroneus brevis reconstruction technique and free hamstring autograft provide good functional results.

**Conclusion:**

The management of chronic ruptures is more demanding than acute tears, because of the retraction of the tendon ends, and the gap makes primary repair impossible. Wound complications and infections are frequent after open procedures. Minimally invasive treatments provide good functional results and lower complications rate.

## 1 INTRODUCTION 

The Achilles tendon is the strongest and largest tendon in the human body, with a tensile strength in the order of 50–100 N/mm [1]. Despite its strength, it is one of the tendons most commonly affected by spontaneous rupture. Most ruptures (75%) occur during recreational activities in men between 30 and 40 years old, in particular in soccer, basketball, tennis, and squash. However, 25% of ruptures may occur in sedentary patients [2]. Even though Achilles tendon ruptures are frequent and are usually not difficult to diagnose for experienced physicians, more than 20% of acute injuries are misdiagnosed, leading to a chronic rupture [3].

A chronic rupture can be defined as a rupture with a delay in diagnosis or treatment for more than 6 weeks [3]. The management of chronic Achilles tendon tears is technically more demanding than the primary repair of acute ruptures, because the tendon ends normally are retracted and the status of the surrounding soft tissues makes primary repair increasingly more difficult [4]. Primary repair is not generally possible, because of the increased gap between the two tendon ends. Different surgical techniques have been described to address this problem. Traditional open surgical options include flap tissue turn down using one [5] and two flaps [6], local tendon transfer [7, 8] and autologous hamstring tendon harvesting [8, 9]. The main concerns of these techniques include complications, especially wound breakdown and infections [10]. These complications are probably related to the scanty soft tissue vascularity, and may require plastic surgical procedures to cover significant soft tissue defects.

However, given the lack of prospective randomized trials and the small size of the studies, a standard procedure is lacking. Less invasive peroneus brevis [11] and flexor hallucis longus (FHL) transfer [12] have been described for the treatment of chronic Achilles rupture. These techniques allow reconstruction of the Achilles tendon preserving skin integrity over the site most prone to wound breakdown. Although not the authors’ preference, good results have been reported with the transfer of the FHL. The FHL is a strong, long tendon and allows bridging of large gaps. The muscle itself is able to produce force that supplements the strength exerted by the gastrocnemius-soleus complex. We prefer not to use this tendon in running and sprinting athletes, who necessitate being able to “grip” the ground in a powerful fashion, and therefore, the loss of flexion of the interphalangeal joint of the hallux would be detrimental to performance. However, in patients when this feature is not of paramount importance, FHL transfer can be used with excellent results [13, 14]. When the tendon gap produced is greater than 6 cm despite maximal plantar flexion of the ankle and traction on the Achilles tendon stumps, the tendon of peroneus brevis is not sufficient to fill the gap, and an ipsilateral hamstring tendon graft is recommended [15].

## 2 CLINICAL PRESENTATION AND IMAGING

The diagnosis of chronic Achilles tendon rupture is more difficult than in acute ruptures. A tendon gap, which is usually palpable in acute rupture, may be absent because of a scar tissue bridge. Active plantar flexion of the foot is usually preserved because of the action of tibialis posterior, the peroneal tendons, and the long toe flexors tendons. Calf muscle weakness, Achilles tendon elongation and a limp can be observed. Specific clinical test can be used to evaluate the Achilles tendon. The calf squeeze test, also known as the Simmonds (in the British Isles) or the Thompson’s (in North America) test, is performed with the patient prone and the ankle clear of the edge of the examination table [16]. The affected leg should be compared with the contralateral healthy leg. The examiner squeezes the fleshy part of the calf, causing the deformation of the soleus and resulting in plantar flexion of the foot if the Achilles tendon is intact. The knee flexion test is performed with the patient prone [17]. The patient is asked to actively flex the knee to 90°. During this movement, the foot on the affected side falls into neutral or dorsiflexion, and a rupture of the Achilles tendon can be diagnosed. A false positive test may occur when there is neurologic weakness of the Achilles tendon. Imaging is useful for the diagnosis of Achilles tendon chronic ruptures. Plain lateral radiographs may reveal an irregular configuration of the fat-filled triangle of Kager. Ultrasonography usually shows an acoustic vacuum with thick irregular edges. T1-weighted MRI shows disruption of signal within the tendon substance, whereas T2-weighted images show generalized high signal intensity.

However, clinical examination is the gold standard. Although the North American literature is full of articles describing the MRI features of such injuries, and there seems to be a predilection for MRI scanning of all these patients; the ultimate examination is accurate clinical examination. More for medico-legal purposes than for clinical indications, we used both MRI and ultrasound scans. However, the ultimate choice of procedure depends on the surgical findings. Given this, imaging in reality is redundant.

## 3 SURGICAL MANAGEMENT OF CHRONIC ACHILLES TENDON RUPTURE

### 3.1 Peroneus Brevis Tendon Transfer

The peroneus brevis tendon transfer is an effective technique to repair a chronic Achilles tendon rupture when the gap between the two tendon stumps is smaller than 6 cm.

**Fig. (1):**
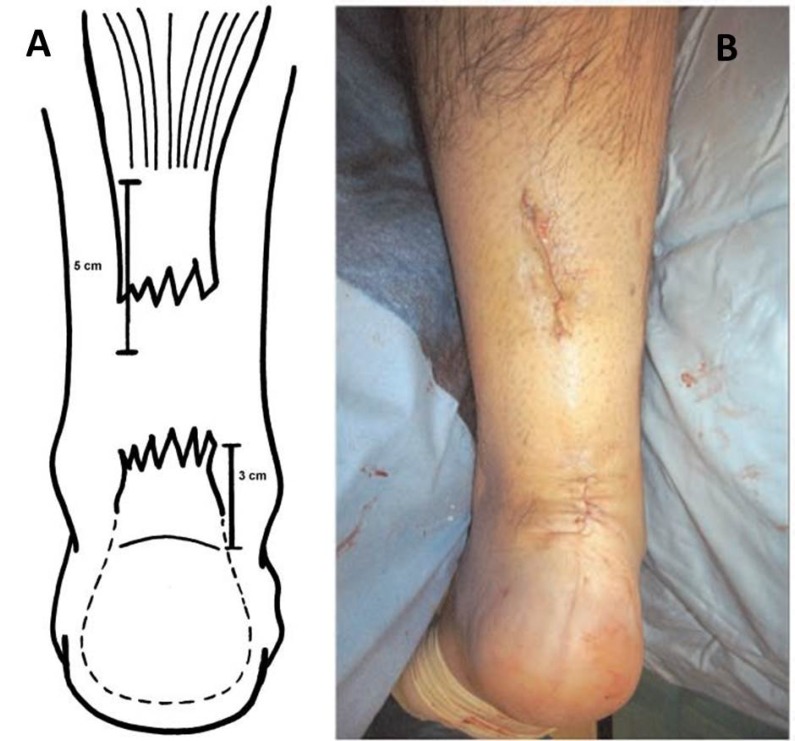
**A)** The first incision is at the proximal end of the palpable tendon stump, as near as possible the medial border of the Achilles tendon. The second incision is lateral to the distal end of the tendon stump. **B)** Final appearance of surgical approaches.

**Fig. (2):**
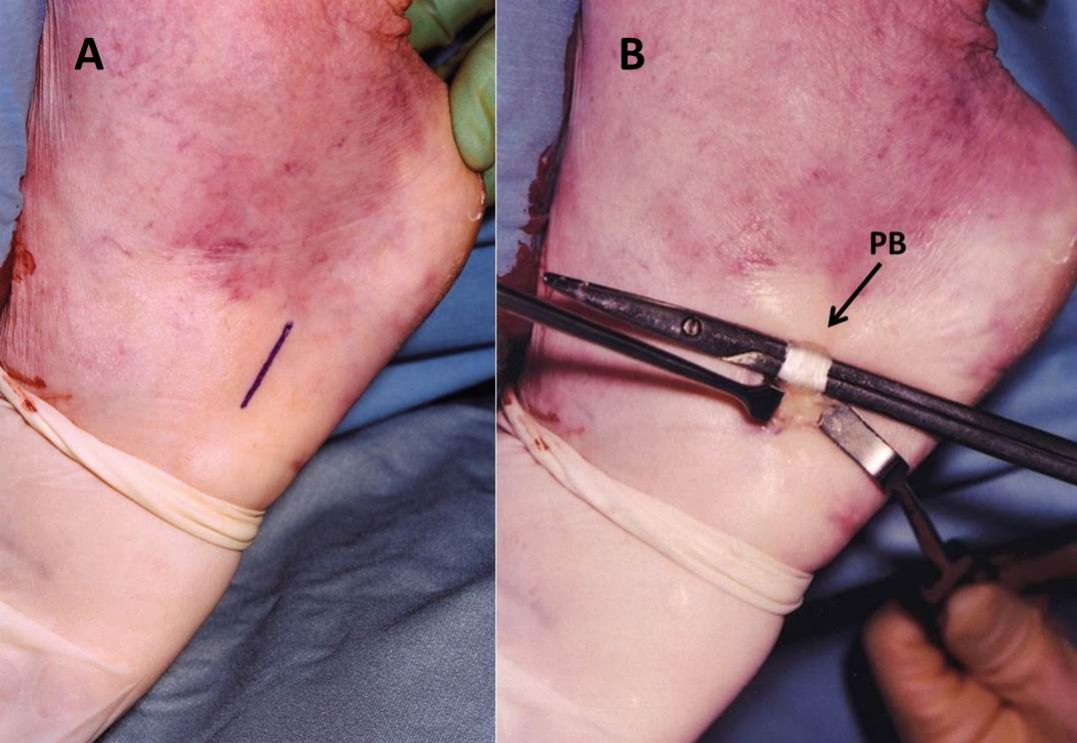
**A)** The third longitudinal incision is over the base of the fifth metatarsal. **B)** The peroneus brevis tendon insertion is exposed and harvested.

The patient is placed prone under general anesthesia, with the ankles clear of the operating table. A tourniquet is applied to the limb to be operated. The limb is exsanguinated, and the tourniquet is inflated to 250 mm Hg. Three longitudinal surgical approaches are made. The first incision is made medially the proximal tendon stump. It is about 5 cm long and it begins 2 cm proximal to the palpable end of the residual tendon. The second incision is lateral to the distal end of the tendon rupture, and it is about 3 cm long (Fig. **1**). Care is taken to prevent damage to the sural nerve by making the incision as close as possible to the anterior aspect of the lateral border of the Achilles tendon. The third incision is a 2 cm longitudinal incision at the base of the fifth metatarsal (Fig. **2**).

**Fig. (3):**
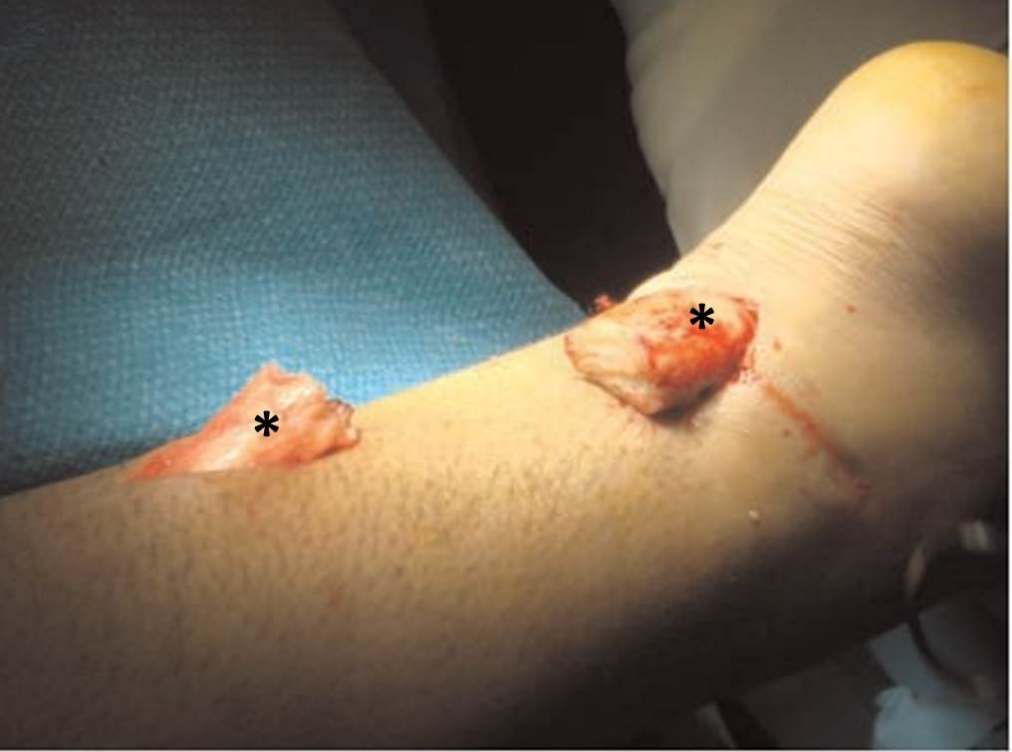
The proximal and distal Achilles tendon stumps are mobilized and exposed. The gap between the two tendon stumps are measured with the ankle in maximum equinus. *: Achilles tendon stumps.

**Fig. (4):**
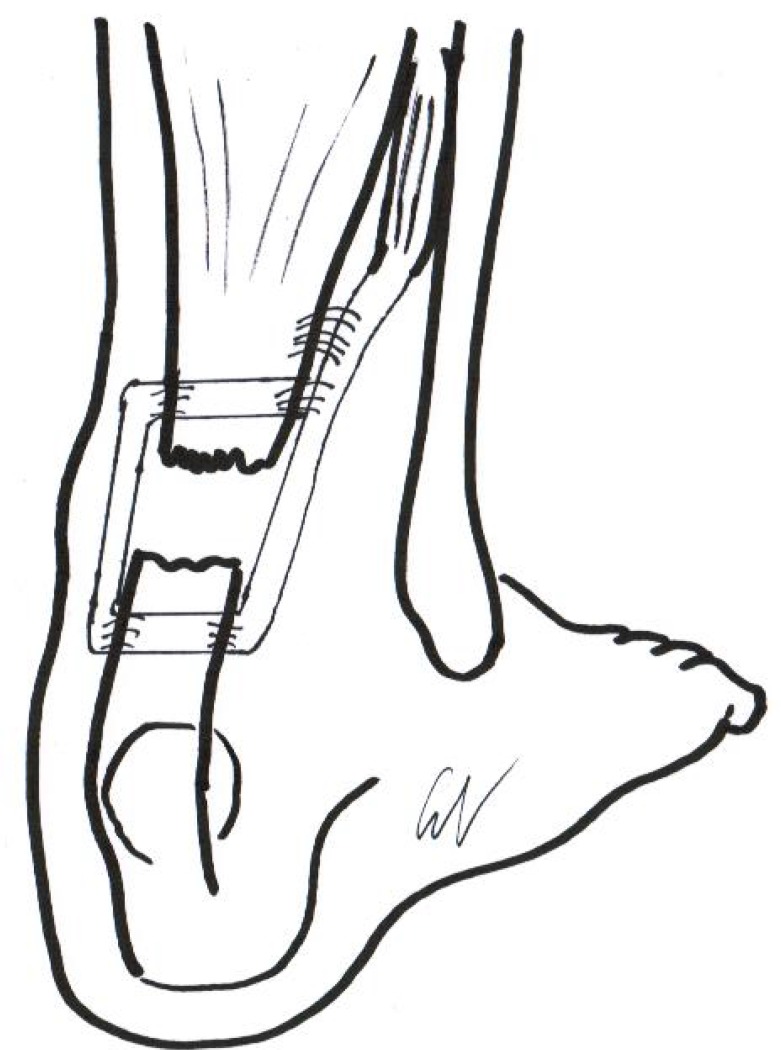
Final appearance of the peroneus brevis tendon transfer.

The distal Achilles tendon stump is mobilized, freeing it of all the peritendinous adhesions, particularly on its lateral aspect. This allowed access to the base of the lateral aspect of the distal tendon close to its insertion. The ruptured tendon end is then resected back to healthy tendon and a No. 1 Vicryl locking suture (Ethicon, Edinburgh) is run along the free tendon edge to prevent separation of the bundles. The proximal tendon is mobilized from the proximal wound, any adhesions observed are divided, and further soft tissue release anterior to the soleus and gastrocnemius muscles allows maximal excursion, minimizing the gap between the tendon stumps. The ankle is then plantarflexed fully and the gap between the stumps is measured (Fig. **3**). The peroneus brevis tendon is identified through the incision on the lateral border of the foot at its insertion at the base of the fifth metatarsal. The tendon is exposed, a No.1 Vicryl locking suture is applied to the tendon distal end, then it is detached from its insertion. The muscular portion of the peroneus brevis is then mobilized proximally to allow increased excursion of the tendon. After incision of the deep fascia overlying the peroneal muscles compartment, the peroneus brevis tendon is withdrawn through the lateral distal incision of the Achilles tendon. This may take significant force as there may be tendinous strands between the 2 peroneal tendons distally. A longitudinal tenotomy parallel to the tendon fibers is made through both stumps of the tendon. A clamp is used to develop the plane, and the peroneus brevis graft is passed through the tenotomy. The graft is passed from lateral to medial in the distal stump of the Achilles tendon. With the ankle in maximal plantar flexion, a No. 1 Vicryl suture is used to suture the peroneus brevis to both sides of the distal stump. The peroneus brevis tendon is then passed beneath the intact skin bridge into the proximal incision and passed from medial to lateral through a transverse tenotomy in the proximal stump; it is further secured with No. 1 Vicryl. Finally, the peroneus brevis tendon is sutured back onto itself on the lateral side of the proximal incision (Fig. **4**). The tourniquet is deflated. The wounds are closed with subcuticular 2.0 Vicryl, and Steri-Strips are applied (3 M Health Care, St Paul, MN), taking care to avoid the risk of postoperative hematoma and to minimize wound breakdown. A below-knee weight bearing cast is applied with the foot in maximal plantar flexion. The mean operative time is about 45 minutes.

### 3.2 Ipsilateral Free Semitendinosus Tendon Graft

If the gap between the proximal and distal stumps is greater than 6 cm despite maximal plantar flexion of the ankle and traction on the tendon stumps, an ipsilateral semitendinosus tendon graft is indicated. The first two longitudinal incisions are the same performed for the peroneus brevis tendon transfer. One incision is medial to the proximal Achilles tendon end, and the second is just lateral to the distal stump, taking care to prevent damage to the sural nerve. Through the proximal incision, the peritendinous adhesions are gently dissected and a partial resection of the proximal tendon stump is performed to expose the healthy portion of the tendon. The free tendon edge is sutured with a #1 Vicryl locking suture to prevent separation of the bundles. The soft tissues anterior to the soleus and gastrocnemius are released to better mobilize the proximal stump of the tendon and minimize the gap. The distal stump is mobilized too. A loop of polyglyconate is used in a Krackow configuration to impose adequate traction to the proximal stump of the tendon. Moderate traction to the proximal stump is applied taking care to maintain the ankle in maximal plantar flexion.

**Fig. (5):**
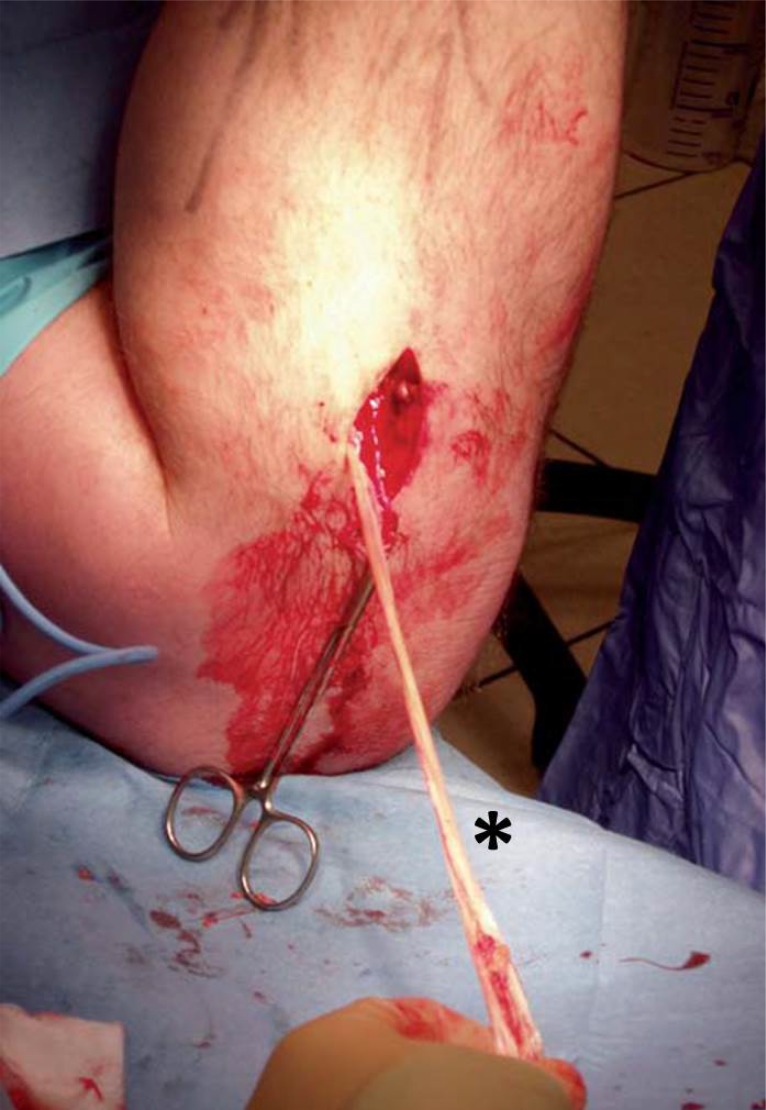
The semitendinosus tendon is harvested with the patient prone, through a vertical, 2.5-3 cm longitudinal incision over the pes anserinus.

The ipsilateral semitendinosus tendon is harvested through a 2 cm longitudinal incision over the anteromedial aspect of the tibia over the pes anserinus [18]. In the original technique, the semitendinosus was harvested with the patient supine, but it was technically demanding, as the patient was upside down, and the anatomy is obviously 'the other way round' [19] (Fig. **5**). Currently, the authors harvest the tendon of semitendinosus with the patient prone, using a 2 cm transverse incision over the palpable tendon of the semitendinosus in the popliteal fossa.

**Fig. (6):**
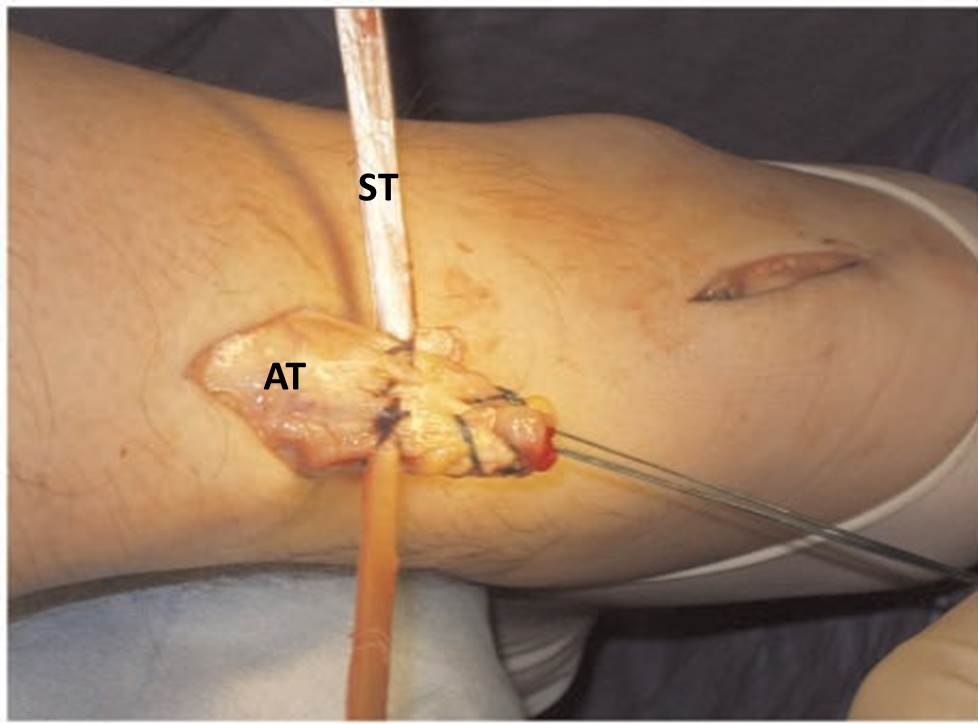
The tendon graft is passed from lateral to medial into the proximal Achilles tendon stump. AT: Achilles tendon; ST: Semitendinosus tendon graft.

**Fig. (7):**
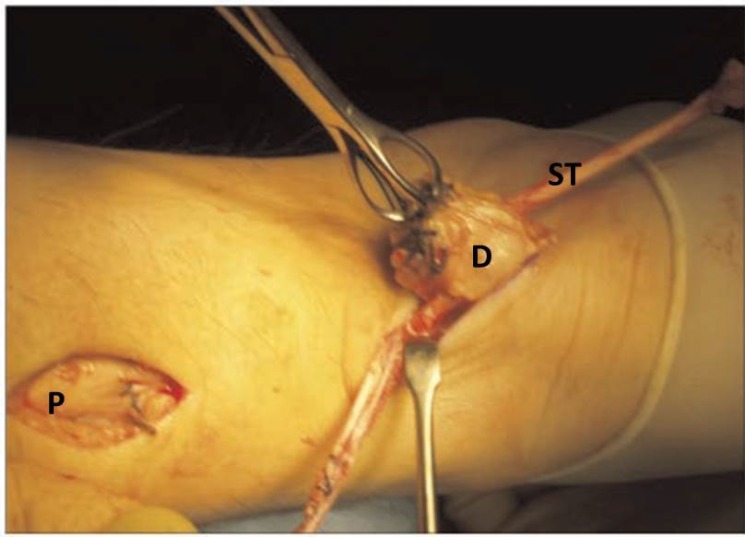
The tendon graft is passed beneath the intact skin bridge from the proximal to the distal incision, and it passed through a transverse tenotomy into the distal stump from the medial to the lateral side. P: Proximal Achilles tendon stump; D: Distal Achilles tendon stump; ST: Semitendinosus tendon graft.

**Fig. (8):**
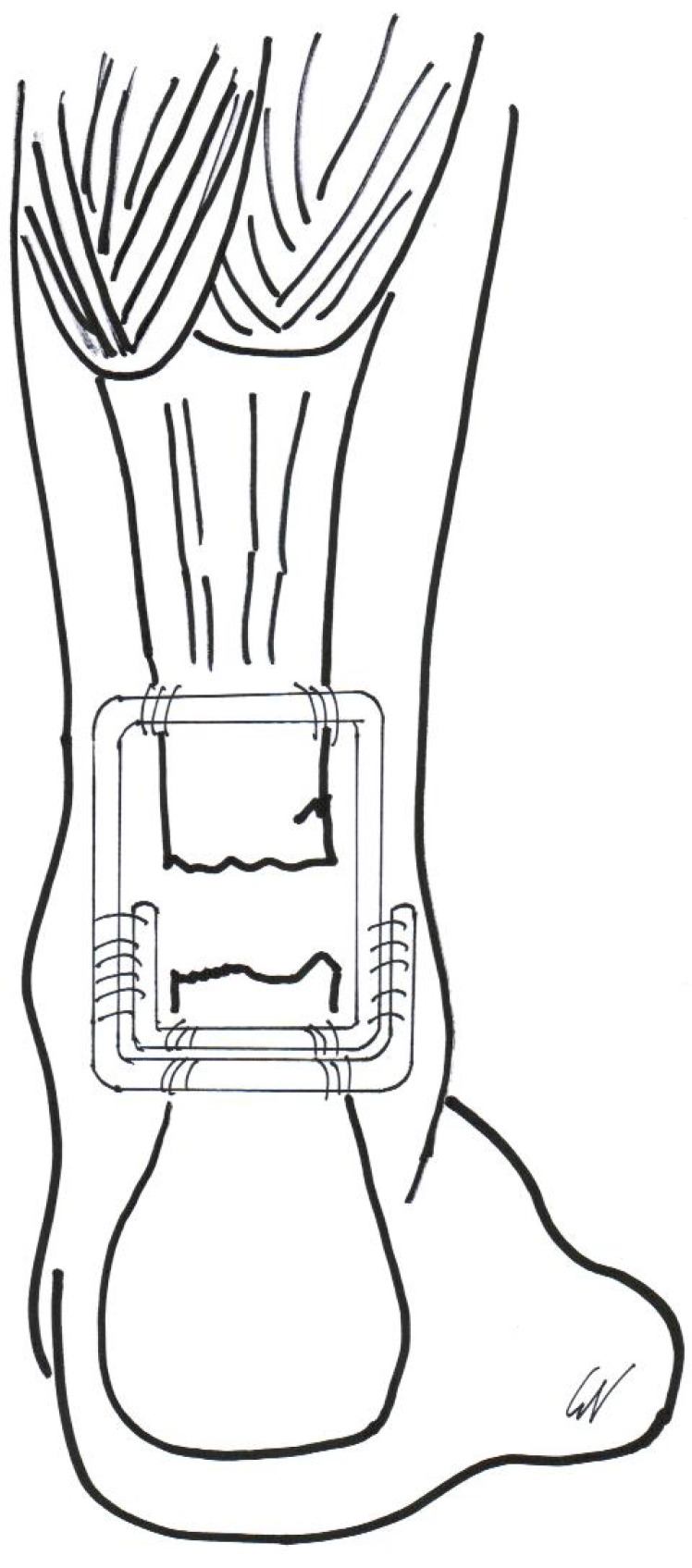
Final appearance of reconstruction of chronic Achilles tendon rupture with semitendinosus tendon graft.

Once the two ends of the semitendinosus graft have been tubularized using a 1-0 Vicryl whipstitch, the graft is passed into the substance of the proximal stump of the Achilles tendon about 2 cm above the tendon end through a small incision, and secured to the Achilles tendon at both the entry and exit points with a 3-0 Vicryl suture (Fig. **6**). Then, the graft is delivered to the distal incision beneath the intact skin bridge and passed through a transverse tenotomy into the distal stump from the medial to the lateral side (Fig. **7**). With the ankle in maximal plantar flexion, the semitendinosus tendon is sutured to the entry and exit points of the distal stump using a 3-0 Vicryl suture, and the reconstruction is tensioned with the ankle in maximum equinus. One extremity of the semitendinosus tendon graft is moved again to the proximal incision, beneath the intact skin bridge and passed into the proximal stump through a transverse tenotomy from medial to lateral. Similarly, the other extremity of the semitendinosus tendon is passed again into the distal stump from medial to lateral (Fig. **8**).

The tourniquet is deflated and, after thorough irrigation with normal saline, the skin incisions is sutured with Vicryl suture and Steri-Strips are applied. A below-knee weight bearing cast is applied with the foot in maximal plantar flexion. The mean surgical time is close to one hour.

## POSTOPERATIVE CARE

Patients are usually discharged on the day after surgery after having been taught to use crutches by a physiotherapist. Thromboprophylaxis is provided. Patients are allowed to weight bear as comfort allows with the use of elbow crutches. However, is important to keep the operated leg elevated as much as possible at home for the first 2 postoperative weeks. The cast is removed after 2 weeks, and a synthetic anterior below-knee slab is applied with the foot in maximal equinus. Patients can graduate to full weight bearing as soon as comfort allows, although full weight bearing rarely occurs on account of balance difficulties. A trained physiotherapist supervises the introduction of gentle mobilization exercises of the ankle, isometric contraction of the gastrocneus-soleus complex, and gentle concentric contraction of the calf muscles. Inversion and eversion of the ankle is also encouraged. At 6 weeks postoperatively, the patient is followed up and the anterior slab removed. Physiotherapists supervise proprioception exercises, and gradual stretching and strengthening exercises. A heel raise is not necessary after removal of the cast. Cycling and swimming are started at 8 weeks postoperatively. Patients are allowed to return to their normal activities at the fifth postoperative month.

## DISCUSSION

Less invasive peroneus brevis transfer and less invasive free semitendinosus tendon graft induce less trauma to the skin around the Achilles tendon, allowing to maintain a sleeve of undisturbed soft tissues around the reconstruction which will benefit the health and healing of the area [20]. The choice of the surgical technique is not based on the patient’s demands, but on the characteristics of the rupture and surgical findings. If the gap is less than 6 cm, a peroneus brevis transfer can easily be performed for Achilles tendon reconstruction, but if the gap is greater than 6 cm this technique is not sufficient to fill the gap, and a semitendinosus tendon transfer is recommend.

Good results have been reported in 32 patients who underwent surgical reconstruction of chronic Achilles tendon tear using peroneus brevis tendon transfer [21]. At final follow-up, all patients were able to walk on tiptoes and to perform at least 10 single legged heel lifts on the affected leg. No patient used a heel lift or walked with a visible limp and they returned to their preinjury working occupation. However, the maximum calf circumference was significantly smaller than the contralateral limb. No reruptures and major complications have been reported. Four patients (12%) experienced a superficial infection which was managed with systemic antibiotics and local dressings. A hypertrophic scar in the area of the Achilles tendon distal surgical wound was reported in 5% of cases. Eversion and plantarflexion was also assessed by Gallant *et al.* [22]. The authors found mild objective eversion and plantar flexion weakness, but subjective assessment revealed no functional compromise. More recently, excellent results have been recently reported in 17 patients [23]. At a mean follow-up of 4.6 years most of patients return to pre-injury sport and daily activities, and 10 of 13 patients who performed sport at the time of injury were able to practice recreational activities. The mean modified Achilles tendon total rupture score improved from 58 pre-operatively to 91 at the time of final review. There were not statistically significant differences in the mean maximum circumference of the calf of the operated limb compared to the pre-operative mean value and to the contralateral unaffected limb. However, the strength of isometric plantar flexion of the gastrosoleus complex and of eversion of the ankle was significantly lower than those of the contralateral limb. In this series, no wound breakdown, infections and reruptures were reported.

Minimally invasive semitendinosus tendon graft showed excellent results in 26 patients with chronic Achilles tendon rupture [15]. The average follow-up was 8.2 years. All patients returned to their preinjury working occupation, and 22 patients returned to their preinjury level of activity at a mean of 6.7 months after surgery. At final follow-up, the maximum calf circumference was significantly higher than postoperatively but significantly lower than the contralateral limb, and the isometric plantar flexion strength in the operated leg was lower than in the uninjured one. However it did not influence the daily activities of the patients. The authors reported rate of superficial infection of 7.6%. One patient (3.8%) developed scar adhesions to the distal wound. No deep infections, wound breakdown, rupture or detachment of the Achilles tendon or other major complications were reported.

The combined use of both semitendinosus and gracilis tendons is not necessary, as a doubled up semitendinosus tendon guarantees optimal length and strength. A doubled up gracilis have been used by the senior Author and favorable long term results of its use to reconstruct in an open fashion chronic tears of the AT have been reported [10, 24]. However, at present the favorite Author’s graft is the semitendinosus, and the use of both semitendinosus and gracils tendons is not advocated [25]. It appears that the clinical and functional results of both techniques are equivalent, and the choice of one graft over the other is only dictated by the length of the gap between the retracted stumps. Obviously, there is no reason why the tendon of semitendinosus could not be used in gaps smaller than 6 cm, but only level 1 studies will be able to ascertain whether one reconstruction technique manifests itself as superior to the other.

## CONCLUSION

Chronic Achilles tendon ruptures have been reported up to 25% of patient with a tear of the Achilles tendon. The diagnosis is more difficult, and management is technically more demanding than primary repair of acute rupture. Complications, such as wound breakdown and infection, are also frequent following open procedures. Minimally invasive reconstruction of chronic Achilles tendon ruptures provides good clinical results, with the advantages of decreased perioperative morbidity and complications rate. Reconstruction with the tendon of peroneus brevis using two para-midline incisions is safe, less invasive, and reliable, and good results have been reported in recent literature. If the tendon gap is higher than 6 cm, a free hamstring autograft is recommended. However, the evidence for using these techniques is inadequate at present. Clearly, studies of higher levels of evidence, including large randomized trials, should be conducted. Future level I trials using validated functional and clinical outcomes, and adequate methodology, are advocated.

## LIST OF ABBREVIATIONS:

AT: = Achilles Tendon
FHL: = Flexor Hallucis Longus
MRI: = Magnetic Resonance Imaging
